# Spatiotemporal pattern discrimination using predictive dynamic neural fields

**DOI:** 10.1186/1471-2202-13-S1-O16

**Published:** 2012-07-16

**Authors:** Jean-Charles Quinton, Bernard Girau

**Affiliations:** 1 Cortex project, LORIA/INRIA Nancy Grand-Est, Université de Lorraine, 54600 Villers-lès-Nancy, France; 2 Pascal Institute / ISPR, Clermont Université, 63177 Aubière, France

## 

Prediction and competition mechanisms are here combined into a neuro-inspired computational model in order to enhance robustness for spatiotemporal tracking and pattern recognition tasks. The research presented in this abstract extends the initial experimental results and mathematical accuracy proof obtained with a single predictor [1] to a set of predictors. This distributed model is grounded on the Continuum Neural Field Theory (CNFT) that uses global inhibition and local excitation to implement competition [2]. External stimulations and internal predictions bias the dynamics of the field so as to constraint the selection and tracking of a target. Conflicting signals are indirectly used to filter out noise and inhibit predictors that are not adapted to the current situation (see Figure 1). The topology of the neural fields grants generalization capabilities to the system, and flexibility is thus further increased as interpolation occurs between predictors.

**Figure 1:**
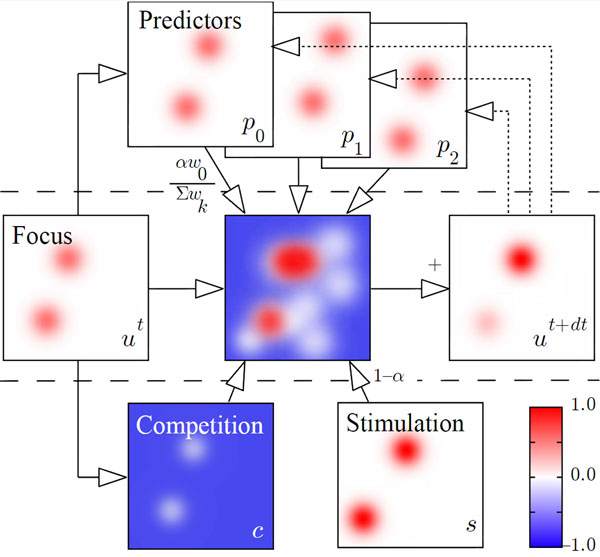
Graphical representation of the extended CNFT equation. The bubble on the focus field will preferentially move where the stimulation and focus activities are strongly correlated, but convergence is here biased by predictors that adequately anticipates the dynamics (p0).

The performance and emergent attentional properties of the model were ascertained on a 2D visual tracking application with ambiguous and noisy signals. Results are synthesized in Table 1, averaged over 60 simulations. A percentage lower than 100% means the performance has improved with an adequate predictor. A value below 20% generally means the original CNFT equation lost the target, in contrast with the extended version.

**Table 1:** Predictive/reactive tracking error ratio

Scenario	Ratio
Competition between distant identical stimuli	114%
Moving target with 30 random distracters	67%
Moving target with Gaussian noise (σ = 0.5)	51%
Obstacle on trajectory (fixed distracter)	10%
Full occlusion of the target after convergence	17%

## Conclusions

While the predictors improve tracking performance when they adequately anticipate the dynamics, their inadequacy simply leads to a fall back on the original CNFT dynamics. This allows the system to perform correctly while learning the predictors, but also to discriminate between trajectories, as the relative level of assimilation of the dynamics is updated in real-time.
